# A life in paleomagnetism: an interview with Lisa Tauxe

**DOI:** 10.1093/nsr/nwag554

**Published:** 2026-08-29

**Authors:** Weijie Zhao

## Abstract

Lisa Tauxe, born in 1956, is a professor emerita at the Scripps Institution of Oceanography, University of California, San Diego. With nearly five decades of dedication to paleomagnetism research, she has established herself as one of the field’s most influential leaders, transforming it into a precise and mature discipline. She was elected to the US National Academy of Sciences in 2015, to the American Academy of Arts and Sciences in 2016, and to the Chinese Academy of Sciences as a foreign member in 2025.

In July 2026, NSR conducted an in-depth interview with Lisa in Beijing. She was warm and cheerful, introducing the fundamental concepts and historical development of paleomagnetism and sharing several fascinating research stories. Even more vividly, she recounted the challenges and setbacks, as well as the heartwarming moments, that she has experienced throughout her own scientific career.

## PALEOMAGNETISM: AN OVERVIEW OF THE FIELD

***NSR:*** Would you briefly introduce the history of the field of paleomagnetism?

***Tauxe:*** People have known about the Earth’s magnetic field since very long ago—the Chinese made the first compass at around 300 BCE.

Then in 1600, an Englishman named William Gilbert, who studied all the then available data about the Earth’s magnetic field, published a book and made the famous assertion that ‘The Earth itself is a great magnet’.

In the late 1800s, people started to think about the possibility of studying the ancient magnetic field by measuring the fired objects, such as ceramics, or volcanic deposits such as lava flows. But it was not until the early 1900s that people actually started such measurements, and unexpectedly discovered that the magnetic field didn’t always point north, sometimes it pointed south. They were very puzzled and it took until the 1960s to prove that the field had in fact reversed.

Since then, the use of paleomagnetic information from rocks for dating has gradually established itself as a scientific approach. I entered this field in the late 1970s. I started with using the fact of reversal to date geological outcrops, and gradually witnessed how paleomagnetism developed into a diverse field over the years.

***NSR:*** How did you enter this field?

***Tauxe:*** When I was 19 and an undergraduate student, I had an opportunity to go to Pakistan for a project. In an area called the Siwaliks in northern Pakistan, people found a lot of fossils about 20–5 million years old, and had been trying to use the directions of the magnetic field to date the rocks.

The outcrops there consisted of alternating layers: one layer of sandstone, one layer of silty clay containing fossils, then the sandstone and then the clay again. My job was to find out whether the sandstone could be used as a single time horizon. Luckily, I found one sandstone that had a magnetic reversal underneath it in one place, then I traced the sandstone

**Figure 1:**
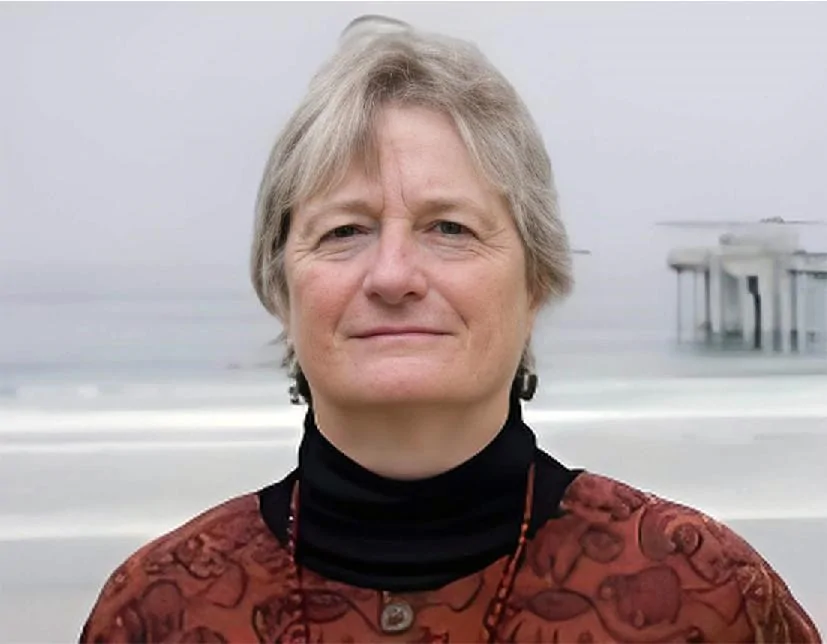
Prof. Lisa Tauxe is one of the most influential leaders of paleomagnetism. (Photo provided by Prof. Tauxe.)

out and followed this reversal by sampling through that sequence over and over again and determined that, yes, these sandstones could be used as a single time horizon.

I also discovered that the magnetostratigraphy interpretation was incorrect for the whole sequence. I showed that if you sample closer together, you may find more opposite intervals and improve the old interpretation. That was my first publication and my first contribution to science.

And then I started wondering, how do these sediments get magnetized? How much time it took and what’s the resolution? How can we use these rocks to study the ancient Earth’s magnetic field? And I think about those same questions still today. I just find them interesting and kept doing it.

***NSR:*** So how did the sediments get magnetized and become a record of the past?

***Tauxe:*** Magnetic fields in crystal structures are generated by the spin of electrons. If the spin directions are random, there will be no overall magnetic field. But in the presence of the Earth’s field, when the material cools down from a very high temperature to what we call the Curie temperature, which

**Figure 2:**
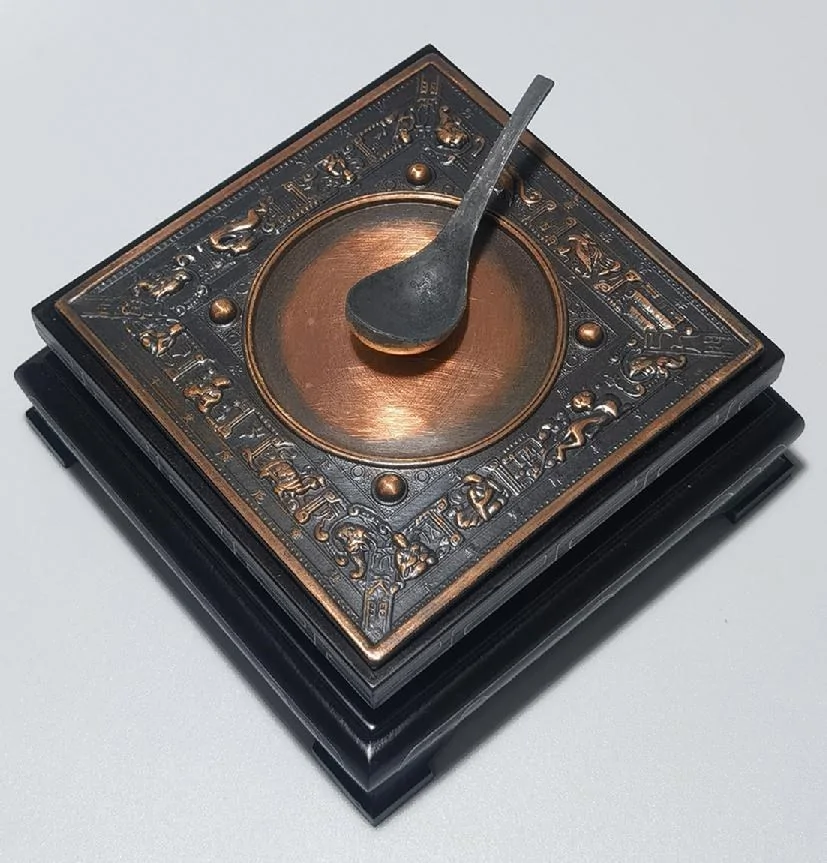
The Chinese compass. (Photo credit: Shuhui Cai.)

is 580°C for magnetite, there would be a slight preference for the spin directions and the material becomes magnetized. As it cools down to room temperature, that magnetization becomes fixed and will stay that way. When igneous rocks get ground up and deposited in rivers, lakes and the ocean, the magnetic minerals have a slight preference for the direction of the magnetic field. So, you have a magnetic record from either lava flows or sediments: it’s in the direction of the magnetic field and the strength is linearly proportional to the magnetic field.

***NSR:*** How do you practically measure the paleomagnetic information?

***Tauxe:*** I use an approach including several steps to obtain reliable results for getting the ancient field strength from volcanic or archaeological objects. The first step is to find a material that will satisfy the requirements of the theory. According to the theory, the samples accurately record the history. But that only works for very small particles, say particles of 30–60 nm or so, because larger particles consist of complex domains and they do not follow the original theory at all. So, my first step is to find fine-grained single-domain materials that will behave like I just described.

Step 2 is to create a laboratory experimental protocol. We need to test whether the sample is actually fine-grained and follows the theory, and whether its magnetization process is reproducible. We need to do three tests: we heat it in small steps of temperature in zero field and measure it, then we heat it in a certain fields, cool it and measure it and then we heat it again in zero field. The first and second tests can tell you whether it follows the theory, and the third one can tell you whether it’s reproducible. This step is what we call ground truthing, and it’s actually more complicated than what I described.

Then you can proceed with the next step: collect your samples and do the experiments.

With the above steps, we may get results with good accuracy. But the next question is the uncertainty. How do you know how many samples do you need to get a good average? How do you determine the uncertainties of the answer? And this is something that I’ve spent a lot of time on.

I’ve been working on the uncertainty problem with my last graduate student Brendan Cych, who was a very clever young man and is a postdoc in France now. He developed, for his graduate thesis, a Bayesian technique called BiCEP (Bias Corrected Estimation of Paleointensity). The method can get a good answer even if the samples are not perfect and with reasonable uncertainty limits. The answers are comparable to the ones from ideal samples.

***NSR:*** Do you need to do all of these steps for every study?

***Tauxe:*** Yes, so it’s a lot of work. Generally, for each study, we need at least 2 weeks to go in the field to sample—and it may take much longer; I spent months in Antarctica. Then back in the laboratory, each measurement takes about an hour and for a set of 50 specimens, it will take totally 3 weeks to get all the data. Then, we need to analyze the data and write the paper. Typically, we need about one year to complete a project.

***NSR:*** How precise are current geomagnetic measurements?

***Tauxe:*** It’s a good question, and under much debate. The intensity of the Earth field depends on where you are. Here in Beijing, it is around 50 micro tesla, and the farther north you go it gets stronger, the farther south you go it gets weaker. Now we can measure the field from a single lava flow with an uncertainty of plus or minus 4 micro tesla. But in more complicated samples, it is probably higher than that, at least double.

***NSR:*** Is the current accuracy good enough to solve the scientific problems?

***Tauxe:*** Well, it depends on which problem. It’s good enough to tell many things. We can tell how long we’ve had a magnetic field somewhere. We can tell that 3000 years ago in Israel, the field was twice as strong as today and even higher. We can tell that 40 000 years ago, the field was much weaker, and whenever the field decides to go from north to south, it gets very low and then comes back up. These things I’m confident in. But if somebody says it’s 50 but not 54 micro tesla—it’s not possible.

## THE SPIKES: HOW FAST CAN THE FIELD CHANGE

***NSR:*** The discovery of the ‘geomagnetic spikes’ from the Levant samples is a major breakthrough. When you first saw the spikes, did you believe it at once?

***Tauxe:*** The story started when a friend of mine in Israel asked me to recover the field intensity from metallurgical slag in Israel and Jordan. People there extracted copper from the ore and dumped the slag for thousands of years. Our analysis showed that the field there was very high 3000 years ago—but that was still not the spike.

Then there was a graduate student named Erez Ben-Yosef. He was a student of an archaeologist but worked in my lab. We went to an archaeological site in Jordan and sampled the pile of slag. Then one day, we were having dinner in his archaeology professor’s house and analyzing the data, and I said, oh my goodness, look, not only is the field really high, but it changed really fast in this outcrop. It went up and down really fast. And that is the spike.

I wasn’t sure if I believed it. At that time, we did not know how fast the field can change. So, what do we do as scientists? We go replicate it.

The first paper was published in 2008, and then we found another outcrop of the similar material across the Jordan River in Israel, and Erez and I along with a new post-doc of mine, Ron Shaar, sampled that and found the same spike, so we reproduced it in two places at the same age.

Then we kept working on this problem after Erez and Ron went back to Israel, and Ron found that there’s not just one spike, but four in a few hundred years. So, the field can change really fast.

**Figure 3:**
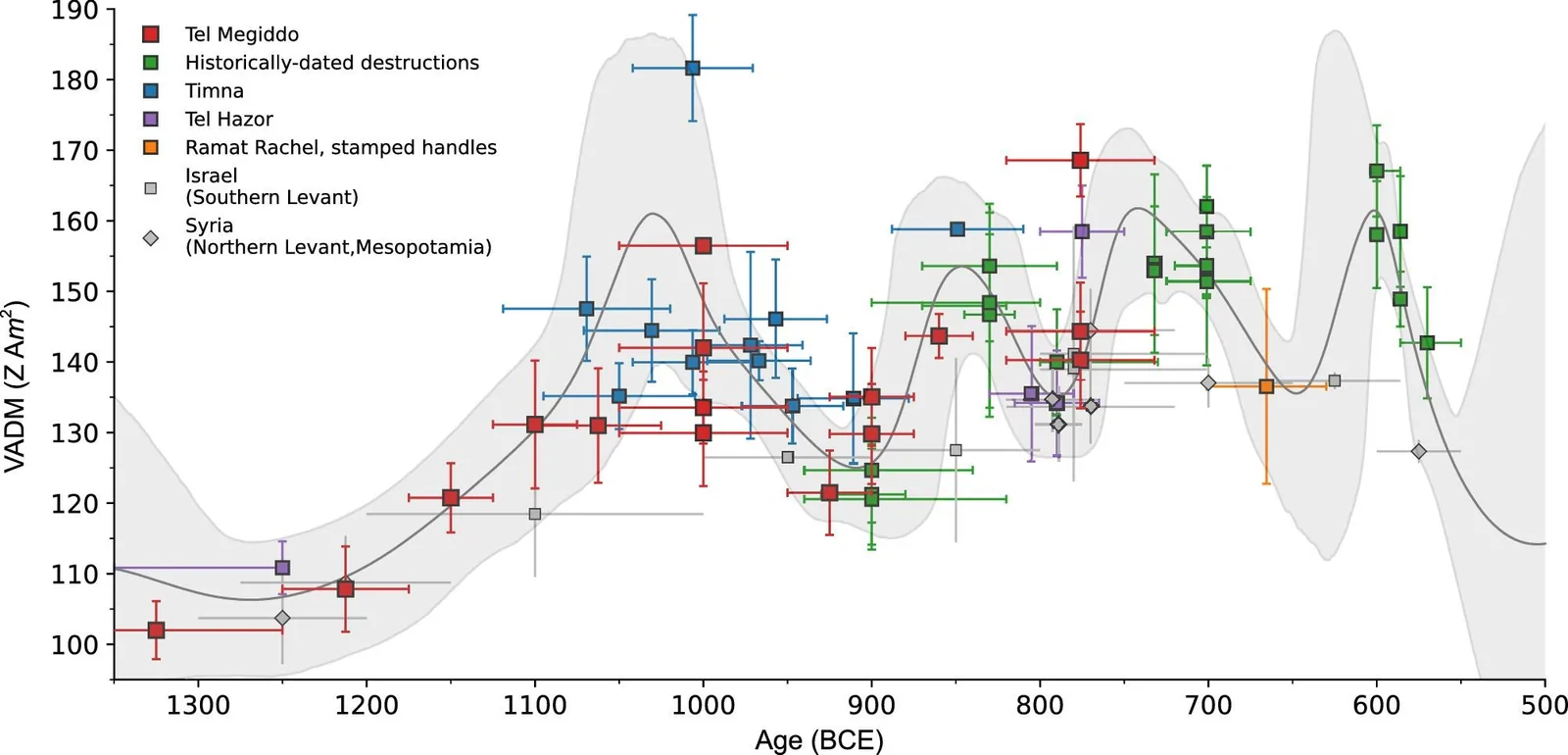
Levantine geomagnetic intensity spikes during the first millennium BCE. (Figure source: Shaar *et al*. (2022), JGR: Solid Earth, Fig. 6a.)

***NSR:*** How did you gradually convince other researchers?

***Tauxe:*** People were very skeptical at first because they do not see the spikes in Europe. But you know, maybe we just haven’t found it yet.

But now we have replicated it many times in many different outcrops, including in China. We still need data in between Israel and China, but unfortunately right now we cannot go to Iran. People are very interested in this field and many papers have been published. Now I do believe it, and I think most people believe it too.

## A DIVERSE AND EXPANDING FIELD

***NSR:*** Can you share one or two examples of your recent works, to tell how the paleomagnetic tools were used to solve real scientific questions?

***Tauxe:*** One thing is that paleomagnetic data can provide a timescale for the paleontological record. Three years ago, I went on a cruise with a graduate student from Oregon State University, Samantha Cargill, to the Greenland margin. The project was to study the ice sheet evolution, and we drilled cores with the US scientific drill ship, which is now retired. Normally people would use the microfossils to date the rocks. But there were none.

So, Sami and I tried to date the core with magnetic field. We worked really hard and even got a record of the field: we measured all the cores, which are each 9 meters long, at 5-cm intervals. We found the last three reversals, which provided three dates; and we bravely produced a timescale for the core relying upon the relative intensity change of the record. We did what we could. I don’t know how accurate it is but I think it makes sense. This work has not been published yet but will come out soon.

Another project I’m working on is that I and some colleagues are trying to answer the question of how long has the Earth’s field been asymmetric about the equator. You know, if the Earth itself is a great magnet, like Gilbert said, then the field should be symmetric. But actually, the field in the Northern Hemisphere is on average stronger than in the Southern Hemisphere. At the Arctic Circle, it’s 10 micro teslas stronger in the north than in the south.

So how long has it been that way? I started a project in 2022 with my student Brendan Cych to address this question for the last 5 million years. I already had samples from Antarctica, and we went to New Zealand, Australia, Hawaii, Iceland, Israel and many other places to get samples from all over the world and over the last 4 million years. Surprisingly, we found that the field stayed stronger in the Northern Hemisphere for the last 5 million years.

The project is still going on and we haven’t published this yet, but I believe our result will be helpful to solve the competing hypotheses on how is the asymmetry generated. Some say it is related to the intensity of field in deep Earth, so that the asymmetry will disappear when the field intensity is low. While others believe the asymmetry comes from big blobs sitting on the core-mantle boundary, and it should always be there. Our work shows that it’s pretty clear that the field was still asymmetric when the field was weaker.

***NSR:*** What are the current major challenges in the field? What are your expectations for the recent years?

***Tauxe:*** One important direction that I am still very interested in is: from a physics point of view, how do rocks get magnetized? Now a team in Liverpool and Edinburgh can model that process with supercomputers. They can model the rocks with complex grains at a level of detail that was not possible even 10 years ago. Two key members of the team were my postdocs, and I am really proud to be able to watch the team grow.

Another interesting thing is what Prof. Yongxin Pan (潘永信) is doing in Beijing. They are discovering the effect of magnetic fields on organisms. He told me yesterday that if you put rats in a zero magnetic field, after a month, they become stupid and cannot solve the simplest problems. This can be recovered after being exposed to a magnetic field again for a while. This is important because if we send people to the moon, where there is no magnetic field, we don’t want to make them stupid. And most of us paleomagnetists spend a lot of time working in magnetically shielded rooms, where the field is not zero but also rather low.

There are also other questions to be answered. How long have we had a magnetic field on the Earth? It seems that we’ve had it ever since the oldest rocks were formed, but what’s the history of the magnetic field over the lifespan of the Earth? Has it changed a lot during that time? Were there times when it decreased to zero? Those are what I’ve been working on, and will keep doing.

## WILL THE EARTH’s FIELD REVERSE AGAIN?

***NSR:*** How much do we currently understand about the mechanisms of the generation and evolution of the Earth’s field?

***Tauxe:*** That is a difficult question and it’s not my field of expertise. We know that the Earth’s crust has some magnetism, but it’s not very strong. Deeper into the mantle, there is basically non-magnetic. Then all the way down to the center of the Earth, the core consists of mostly iron, a little bit nickel and some other things. The outer part of the core is molten, while in the very center it is solid. The fluid outer

I have a bet that there will not be a reversal in 500 years.

—Lisa Tauxe

core is convecting up and down, and the Earth is spinning day and night. Such convection generates the so-called Taylor columns, which I believe is the source of the Earth’s field.

Detailed description of the mechanism requires supercomputers to solve seven nonlinear equations simultaneously, which is so complicated that it’s far beyond my skill and maybe beyond the ability of the supercomputers.

***NSR:*** So, we are still far from being able to predict the evolution of the Earth’s field?

***Tauxe:*** I don’t think we ever will be able to, because the system is nonlinear and chaotic. It is not only very difficult, but maybe theoretically impossible.

***NSR:*** Do you think there will be big changes, such as another reversal, in the near future?

***Tauxe:*** I have a bet that there will not be a reversal in 500 years. The field is now going down, and some of my colleagues believe that this is a sign of the next reversal, but I don’t think so, because the field has always been going up and down, and the current field strength is still double of the historical average level. In the last 800 000 years, the field has gone up and down many times but only once did it try to flip, at about 40 000 years ago, but it didn’t make it.

But the decrease of the field may affect our infrastructure. If it becomes too low, it might not be able to protect us from solar and cosmic radiation, especially when there’s a solar coronal mass ejection, which happened recently. Our satellites and electrical grids may be affected.

***NSR:*** What will happen when the field does flip? How will it influence human or animals?

***Tauxe:*** This is an interesting question, and the answer is that there’s very little influence on animals.

Early on, there was an argument that the dinosaurs were killed off because the magnetic field flipped. But that’s not true. The dinosaurs died out right in the middle of two flips, so the field reversal did not cause that extinction—an asteroid did.

But there are always repeated arguments that reversals have somehow caused some extinctions. Some Chinese scientists say that at the end of a very long reverse period, there was an extinction event related to the very low field. And some of my friends say that the attempt to flip 40 000 years ago was related to the extinction of the Neanderthals. It is possible, but—I don’t know.

**Figure 4:**
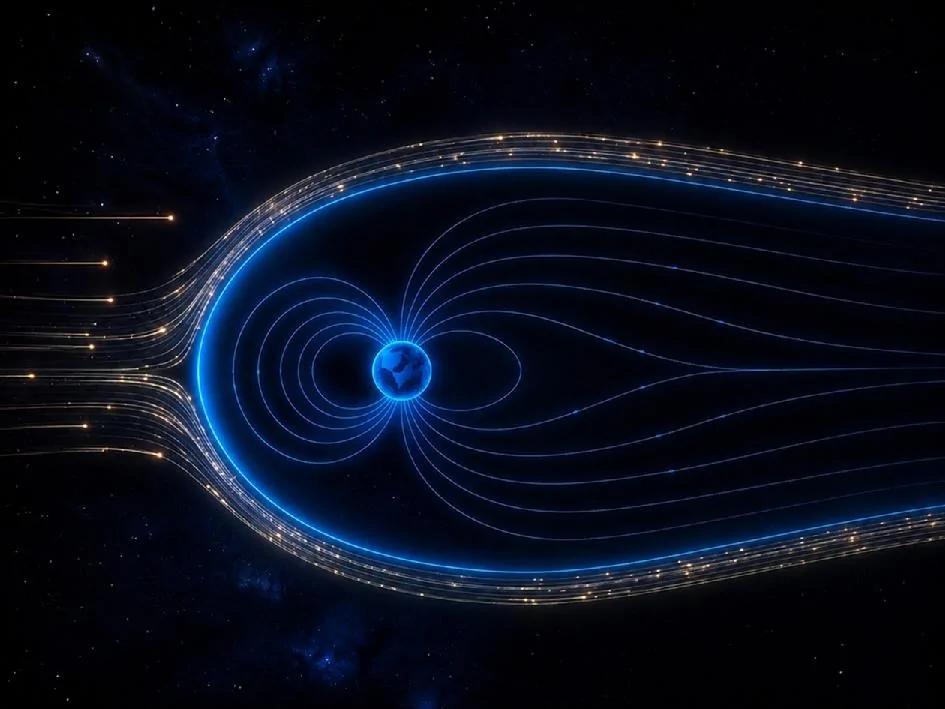
Earth’s magnetosphere deflecting energetic particles from the solar wind. (Image generated by Yuhang Zhao with the help of ChatGPT (GPT-5.6 Sol), OpenAI.)

## OPEN SCIENCE AND OPEN PUBLICATION

***NSR:*** We know you are a supporter of ‘open science’. In the process of promoting data and knowledge sharing, have you encountered some challenges?

***Tauxe:*** Yes. We created the MagIC (Magnetics Information Consortium) database. And as the chair of the publication committee of the American Geophysical Union (AGU), I supported the requirement that all the authors who publish in our journals make their data openly available in a findable, accessible, interoperable and reusable permanent database.

Many people do put their data in there, but they also complain and hate it because it is too complicated to get all the metadata correct. Our experiments are complicated, so you need to keep track of many things: when was the measurements, what was the field strength, what direction was the field—all these metadata. So, the data tables have many columns. And when people look at the data in detail, they said: but you forgot this one. So, we have to add another column and to make it even more complicated.

This has been an evolving process over the last few decades, and it requires great patience and encouragement for us to help people to get their data into the database. But once it’s in the database, then it is findable, accessible, interoperable and reusable, and people are actually reusing the data happily all the time. I think people are getting happier with the idea now.

***NSR:*** What do you think about open publication, including the preprint, open access publication and open peer review?

***Tauxe:*** Open peer review is a good question. As a reviewer myself, I usually sign my reviews, but not always. I try to think, will this person listen to me better if they don’t know it’s me? You know, sometimes if they know it’s me, they may say, oh, of course she would say that and I will not listen. So, although I think open peer review has its advantages, I would not like to be forced to always reveal who I am.

Then the preprints. I think they’re very useful as long as everyone knows this is not the reviewed version. It’s very useful to get things out there. I know the medical community uses it a lot and our Earth science community is increasingly doing so as well.

Open access publication has now become mainstream, and the issue is who pays, the libraries or the authors? The AGU journals charge the authors around $3000 for one publication. Most scientists can probably afford that, but what about if you come from Africa and have no funding? Running a journal costs money, and the staff have to get paid. So, it is reasonable to charge the fee, but the price has to be affordable.

## LISA’s CAREER STORY

***NSR:*** You’re American, but you went to high school in Switzerland—how did that happen?

***Tauxe:*** My dad was a doctor and he had a patient who sent his kids to the school—a little school named Ecole d’Humanité in Switzerland on a mountaintop. And when I was 14, my father took us around Europe for a tour. We visited the school and talked to the teachers there. My mom loved it. She thought it was fantastic.

Then when I was 16, my family moved from Minnesota to Alabama, which is in the South and is very conservative. The schools were not very good there—there was a good boy’s school, but they didn’t take girls. My mother was worried that I might get into trouble because I have a big mouth—I say whatever I think. So, they decided to send me to that school in Switzerland. It was a wonderful school. It was a small community with no grades and gave me a great amount of freedom to study what I wanted to. I spent 2 years there, and it was perfect for me.

***NSR:*** Have you personally encountered any obstacles related to gender or background in your career?

***Tauxe:*** Yes, of course. In the early 1980s, I married a guy who studies volcanoes. He’s very lovely and supportive. He and I joined our institution, Scripps Oceanography, in 1983. I got a research position, and he was a postdoc, then he got a research position and I became a professor.

And when he was going to get his formal job, we were a little worried. At the time, we got job offers from another university for two professorships. It happened to be in the town where my parents lived. During that period, we had one little boy and then another little boy, so that would have been nice if we went there.

Then I went to our Dean and said, we have two job offers here for two professorships; should we take them or will Hubert get his promotion and then we can stay here? He said Hubert is fine; his file is almost through and he’s going to get

I went to the Dean and asked why. He said, Lisa, you don’t need a job because your husband can support you.

—Lisa Tauxe

the promotion. I asked, can I have that in writing? He said, why don’t you trust me? And I did.

But a few months later, the committee lost his file and created a new one. And somebody else was in charge, and she, who never had kids, thought I was being silly to be the first mother on the faculty. I had tenure and she couldn’t fire me, but she fired Hubert.

So, we started looking for jobs. Hubert got a really good job offer in the Netherlands, and there was also a paleomagnetism lab there, run by a guy who was very old and was about to retire. We thought maybe I can get that job when he retires—I was doing pretty well at that time.

So, we went there and spent 4 years in the Netherlands. We raised our little boys and I learned Dutch. I built the magnetic shielded room with some help from the lab technician. I designed it and built it. That magnetic shielded room had the lowest field of any lab in the world at the time. But when the old man retired, they gave the lab to a former postdoc of mine, and I was left with nothing. They even took away my position where I could write grants and recruit students.

I went to the Dean and asked why. He said, Lisa, you don’t need a job because your husband can support you. I asked, so how do I write my grants? The guy said, you can write the proposal and the new director of your lab, who was my postdoc, can submit it for you in his name. And I said, wow.

I went home to my husband and said, it seems that there’re only three opportunities for jobs for me here in this country, prostitution, selling drugs or alcoholic housewife—so which of these three do you think is the best option for me? Then in the Netherlands, only 3% of moms had full-time jobs—maybe it is better now. You know, that was just pure sexism, had nothing to do with my abilities.

In the end, we were able to go back to Scripps and we could finish our careers there. But yes, I did have a little bit of a detour there, which was clearly based on gender.

***NSR:*** Do you have any particular interesting experiences during your collaboration with Chinese scientists?

***Tauxe:*** I came to Beijing for the first time in 2007, the year before the 2008 Beijing Olympics. Since then, I’ve been coming every few years.

The second time I came here, around 2010, I gave a talk at the Institute of Geology and Geophysics, Chinese Academy of Sciences (IGGCAS) about that spike, which was just discovered then. At the end of the talk, I said, China, like Israel, is rich in ancient archaeological sites, and I think you should do some archaeomagnetic studies; there were a series of papers published in the early 1980s by IGGCAS Professor Qingyun Wei (魏青云), but nobody followed up.

At the end of the talk, a petite young lady walked up to me. She looked at me and said, ‘That is what I want to do!’ I cannot forget that big smile on her face. That was Shuhui Cai (蔡书慧). She was then a graduate student. Later, Prof. Rixiang Zhu (朱日祥) kindly sent her to my laboratory for a year to work on Chinese archaeomagnetism, and she brought the field back to life. Then she came as a postdoc for 3 years in my lab, and now she is back in IGGCAS as an established scientist. We had many adventures together and established long-term friendship and collaboration. Now, I have begun to work with her graduate students.

***NSR:*** Do you have any suggestions for paleomagnetism research in China?

***Tauxe:*** There are several excellent Chinese groups, including the IGGCAS groups led by Profs. Zhu and Pan, Qingsong Liu’s (刘青松) group in Shenzhen and the Peking University group led by Baochun Huang (黄宝春) and Liao Chang (常燎). These people are working very hard and have very good ideas.

Paleomagnetism is a relatively small field, but it is flourishing in many areas around the world—in China, in Europe and even in Mexico and Brazil.

***NSR:*** What do you think are the most important qualities for becoming an excellent Earth science researcher?

***Tauxe:*** I think there are three. First, you need curiosity. You need to wonder about things and come up with ideas or hypotheses. Then you need the willingness to work hard to realize your ideas. And the third and maybe most important is persistence. Facing difficulty, you should not give up; keep on and go find another way. I think these three traits are what I look for in students.

## Notes

Weijie Zhao is an NSR news editor based in Beijing.

